# Uterine Contractility Changes in Adenomyosis: Evidence from a Systematic Review and Meta-Analysis

**DOI:** 10.3390/biomedicines13112728

**Published:** 2025-11-06

**Authors:** Angela Vidal, Paula Tepasse, Vithusha Vinayahalingam, Sophie Cottagnoud, Marietta Gulz, Tanya Karrer, Gürkan Yilmaz, Janna Pape, Michael von Wolff

**Affiliations:** 1 Division of Gynecological Endocrinology and Reproductive Medicine, Women’s University Hospital, Inselspital Bern, University of Bern, 3010 Bern, Switzerland; sophiemanon.cottagnoud@insel.ch (S.C.); janna.pape@insel.ch (J.P.); michael.vonwolff@insel.ch (M.v.W.); 2 Faculty of Medicine, University of Bern, 3008 Bern, Switzerland; paula.tepasse@gawnet.ch; 3 Department of Gynecology and Obstetrics, Hospital Wolhusen, 6110 Wolhusen, Switzerland; vithusha19@hotmail.com; 4 Department of Gynecology and Obstetrics, Bern University Hospital, University of Bern, 3010 Bern, Switzerland; marietta.gulz@insel.ch; 5 Medical Library, University Library of Bern, University of Bern, 3012 Bern, Switzerland; tanya.karrer@unibe.ch; 6 Centre Suisse d’Electronique et de Microtechnique CSEM SA, 2002 Neuchâtel, Switzerland; guerkan.yilmaz@csem.ch

**Keywords:** uterine peristalsis, uterine contractility, adenomyosis, fertility

## Abstract

**Background**: The presence of ectopic endometrial glands within the uterine myometrium in patients with adenomyosis has been associated with adverse fertility outcomes. UP (Uterine Peristalsis), a movement of contractions at the junctional zone of the non-pregnant uterus, can be impacted by an altered architecture of uterine layers. Abnormal contractility patterns could impact both uterotubal sperm transport as well as embryo implantation. Because of this potential influence on clinical symptoms and reproduction in patients with adenomyosis, studies have been analyzing the feasibility of diagnostic techniques in assessing uterine peristalsis. **Objective**: This systematic review and meta-analysis aimed to detect an alteration in patterns of UP in patients with adenomyosis. **Methods**: A systematic literature search of Medline, Embase, Cochrane, CENTRAL databases and Google Scholar was conducted up to June 2025, including studies evaluating UP and adenomyosis. Clinical studies evaluating uterine contractility were included, excluding those potentially affected by therapeutic interventions. The meta-analysis pooled data from studies to compare uterine contractility direction between patients with adenomyosis and control groups. **Results**: In seven included studies (442 women), uterine contractility varied significantly in association with menstrual cycle phases and pathological conditions. The meta-analysis revealed two statistically significant findings: women with adenomyosis showed significantly reduced uterine contraction frequency (SMD −1.81, 95% CI: −3.04 to −0.58, *p* = 0.0039) and fewer antegrade contractions (OR 0.35, 95% CI: 0.13–0.96, *p* = 0.0423) compared to controls. Other contractility patterns showed non-significant trends with substantial heterogeneity. **Conclusions**: Our findings show a significant difference in uterine contraction patterns in patients with adenomyosis compared to controls, namely a decrease in frequency and an increased number of retrograde uterine contractions in the adenomyosis group. The remarkable heterogeneity of the results highlighted the need for larger study cohorts in the future, especially to address the main diagnostic possibilities and treatments in order to improve reproductive outcomes.

## 1. Introduction

Adenomyosis is a benign gynecological disorder characterized by the presence of endometrial epithelial cells and stromal fibroblasts abnormally located within the myometrium, where they induce hyperplasia and hypertrophy of the surrounding smooth muscle cells [1,2]. The prevalence of adenomyosis in women of reproductive age is estimated to be between 20 and 35% [1]. However, adenomyosis can be asymptomatic in approximately one third of cases. Within the remaining two-thirds of cases, the most prevalent symptoms are menorrhagia (50%), dysmenorrhea (30%), metrorrhagia (20%) and adverse reproductive outcomes [3,4]. Additionally, adenomyosis often coexists with endometriosis [4,5,6].

The impact of adenomyosis on fertility and the success of in vitro fertilization (IVF) has been a subject of debate. A recent meta-analysis showed that adenomyosis is associated with adverse fertility outcomes such as significantly lower clinical pregnancy rates (CPR) and higher miscarriage rates after assisted reproductive technology (ART) treatments [7,8]. These adverse fertility outcomes may be linked to changes in the uterine peristalsis pattern in individuals with adenomyosis. In addition to altered uterine peristalsis, other mechanisms may contribute to subfertility in adenomyosis, including chronic inflammation, impaired endometrial receptivity due to progesterone resistance, and structural or vascular changes within the junctional zone. Coexisting endometriosis may further compromise implantation and pregnancy outcomes.

Uterine peristalsis (UP) has been defined as a wave-like movement that occurs continuously throughout the menstrual cycle in the uterine junctional zone of the non-pregnant uterus [9,10,11,12]. Notably, the active transport of sperm is a crucial aspect of fertility, underscoring the significance of embryo implantation for reproductive success [13]. Abnormal contractility arising from alterations in the architecture of the JZ myometrium has the potential to impact both embryo implantation and sperm transport, ultimately leading to impaired fertility outcomes [11]. Interestingly, at the cellular level, morphological alterations are associated with adenomyosis. A recent study by Mehasseb et al. has demonstrated significant ultrastructural modifications in smooth muscle cells of adenomyotic uteri, characterized by cellular and nuclear hypertrophy when compared to cells in the eutopic myometrium [14]. Alterations of this type have been associated with the development of uterine contractile dysfunction, a condition that has been shown to exert a detrimental effect on uterotubal transport [11]. Additionally, the loss of nerve fibers in the transitional zone between the endometrium and the myometrium, which is also linked to adenomyosis, could contribute to the alteration of uterine peristaltic activity [15].

Furthermore, although conditions such as adenomyosis are increasingly recognised for their association with altered contractility of the uterine junctional zone, current diagnostic approaches remain limited and inconsistent [16]. A potential contributing factor to this variability is the utilization of differing diagnostic criteria and imaging modalities when evaluating both adenomyosis and UP [3,5,17]. Despite the fact that UP has been the focus of research since the 1990s [11], the available data remain heterogeneous and have yet to be widely incorporated into clinical practice.

Notably, the heterogeneity of the measurement tools employed across studies constitutes a primary contributing factor to this inconsistency, as there exists no universally accepted reference standard to date. Invasive techniques, including intrauterine pressure catheters and electrohysteroscopic electrodes, have demonstrated a degree of reliability that is largely attributable to their capacity to interfere with the natural physiology of the uterus, thereby altering peristaltic activity [14]. Conversely, transvaginal ultrasound has emerged as the prevailing modality, attributable to its accessibility, advancements in ultrasound technology, and the development of dynamic assessment techniques. These techniques encompass visual and computer-assisted analysis of cine loops, as well as Cine-MRI [18,19,20,21].

This systematic review aimed to investigate the impact of adenomyosis on altered patterns of uterine peristalsis which, in turn, might have a great influence on sperm transport and embryo implantation, which ultimately affects clinical pregnancy rate. Furthermore, it should provide insights into the pathophysiology of adenomyosis and its effects on reproductive outcomes.

## 2. Materials and Methods

### 2.1. Registration of Protocols

**Figure 1:**
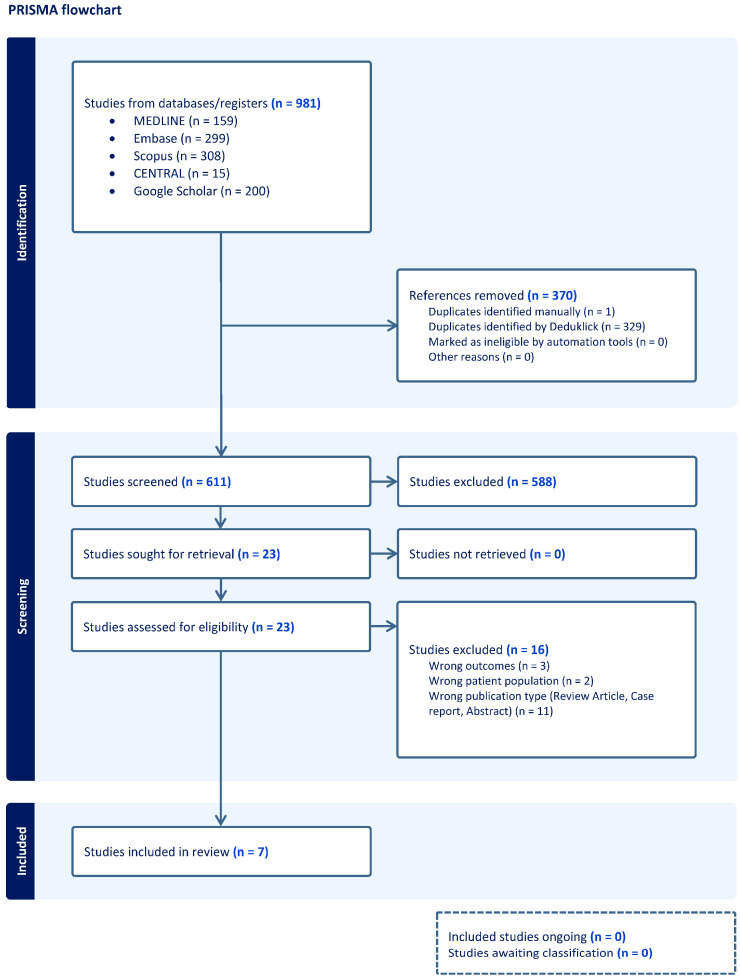
PRISMA flow chart. Flowchart of the bibliography search and selection process.

The protocol was registered in the Prospective International Registry of Systematic Reviews, PROSPERO (registered number CRD420251107580). The guidelines for the Preferred Reporting Items for Systematic Reviews and Meta-Analyses (PRISMA) have been used [22] (Figure 1).

### 2.2. Search Strategy

A systematic literature search was conducted using the Medline, Embase, Cochrane, and CENTRAL databases as well as Google Scholar in June 2025. A specialized librarian developed an initial Embase search strategy and tested a basic reference list. Following refining and querying, complex search strategies were developed for each information source based on database specific controlled vocabularies (thesaurus terms/headings) and text words. Synonyms, acronyms and similar terms were included in the text word search. Publications from 1946 to the present were included in the search. The following search terms were included: ‘uterine contractions’, ‘uterine peristalsis’, ‘junctional contractions’ and ‘adenomyosis’. Animal-only studies were excluded from all database searches using a double-negative search strategy based on the Ovid “humans only” filter [23]. The detailed final search strategies are provided in the Supplementary File S1. Reference lists and bibliographies of relevant publications were screened for relevant studies in addition to the electronic database searches. All identified citations were imported into Covidence.

### 2.3. Inclusion and Exclusion Criteria

Two reviewers (AV and VV) independently screened all titles and abstracts for eligibility. Studies were included if they were original prospective investigations evaluating uterine contractility in women with a confirmed diagnosis of adenomyosis compared with healthy controls. Diagnosis had to be based on standardized imaging criteria (transvaginal ultrasound or cine-MRI) or histological confirmation. We excluded studies involving hormonal or therapeutic interventions that could alter uterine peristalsis (e.g., GnRH agonists, oral contraceptives, progestins, levonorgestrel-releasing intrauterine devices, or ovarian stimulation protocols), studies with inadequate design, animal studies, and non-original publications (reviews, letters, or conference abstracts). In cases where overlapping populations were identified, the study with the largest sample size or the most complete dataset was included to avoid duplication.

### 2.4. Data Extraction

The extracted data were independently summarized by AV and VV and were the subject of a detailed review (Table 1 and Table 2). The primary variables of interest included characteristics of the study populations such as patient age, cause and duration of infertility, parameters related to uterine contractility such as time of measurement and number of contractions per minute. Disagreements were discussed and resolved by consensus.

### 2.5. Quality Assessment

The Newcastle-Ottawa scale (NOS) was utilized to evaluate the quality of the individual studies [24]. Three parameters were considered for individual study scoring: subject selection (0–4 stars), comparability (0–2 stars), and study outcome (0–3 stars). The scoring was composed as follows: Good quality (=3 or 4 stars in the selection domain AND 1 or 2 stars in the comparability domain AND 2 or 3 stars in the outcome/exposure domain), fair quality (=2 stars in the selection domain AND 1 or 2 stars in the comparability domain AND 2 or 3 stars in the outcome/exposure domain), and poor quality (=0 or 1 star in the selection domain OR 0 stars in the comparability domain OR 0 or 1 stars in the outcome/exposure domain). All included studies were reviewed by investigators AV and VV to independently assess risk of bias. Disagreements were resolved by consensus. Scoring was conducted according to the terms listed in Table 3.

**Table 1:** Characteristics of included studies. Summary of cohort studies investigating uterine contractility and adenomyosis.

First Author, Year of Publication	Study Design	Country	Number of Participants (Study Group)	Number of Participants (Control Group)	Total Number of Participants	Age (y), Mean or Median ± SD (Study Group)	Age (y), Mean or Median ± SD (Control Group)	Treatment Cycle	Moment of Contractility Measurement	UP Measurement Method	Uterus Dimensions, mm (Study Group)	Uterus Volume, cm^3^ (Study Group	Uterus Dimensions, mm (Control Group)	Uterus Volume, cm^3^ (Control Group)	Endometrium Thickness, mm
Kissler et al., 2006 [25]	Prospective observational study	Germany	35 (24 focal and 11 diffuse)	6 (only endometriosis)	41	focal: 32.4 ± 2.6; diffuse: 35.2 ± 4.3	33.2 ± 2.9	natural	NR	HSSG	NR	NR	NR	NR	≥9 mm
Kissler et al., 2007 [26]	Observational cohort study	Germany	Review					NR	NR	NR	NR	NR	NR	NR	NR
Soares et al., 2023 [27]	Prospective case–control study	Brazil	4	11	15	NA	NA	NR	NR	MRI	NR	NR	NR	NR	NR
Arena et al., 2024 [28]	Prospective observational study	Italy	18	18	36	34.6 ± 6.1	33.3 ± 5.9	natural	periovulatory phase (11–14 d) Graafian follicle >18 mm, trilaminar endometrium ≥7 mm	TVS (2D)—reconstruction of coronal plane using 3D software	(33.3 ± 5.9) × (46.9 ± 16.8) × (59.2 ± 15.1)	137.5 ± 117.7	(75.3 ± 9.5) × (37.2 ± 6.2) × (49.6 ± 7.6)	74.5 ± 27.6	≥7 mm periovulatory phase
Rees et al., 2024 [29]	Multicenter prospective study	The Netherlands, Italy, Greece	39	106	145	38.23 ± 7.46	29.4 ± 6.74	natural	menses, midfollicular, late follicular, early luteal, late luteal	TVS	(81.1 ± 14.1) × (49.1 ± 9.9) × (55.0 ± 13.4)	114.67 ± 36.89	(59.1 ± 21.5) × (31.7 ± 10.3) × (40.9 ± 19.9)	40.12 ± 24.34	study group Menses 4.68 ± 2.68 Midfollicular 5.23 ± 2.54 Late follicular 6.65 ± 3.56 Early luteal 10.00 ± 0.00 Late luteal 10.35 ± 3.43 control group Menses 3.50 ± 0.71; Midfollicular 6.50 ± 0.71; Late follicular 5.67 ± 2.08; Early luteal 8.67 ± 0.58; Late luteal 7.33 ± 0.58
Kido et al., 2025 [30]	Prospective observational study	Japan	182 walls (Diffuse 84 and focal 98)	96 walls	139	NR	NR	natural	proliferative, luteal, menstrual	MRI	NR	NR	NR	NR	NR
Latif et al., 2025 [31]	Inter- and intra-observer reproducibility study	UK	26	40	66	39 (35–43)	36 (29–40)	ART	baseline, ovarian stimulation, embryotransfer	TVS	NR	NR	NR	NR	NR

Abbreviations: SD (Standard Deviation), NR (Not Reported), HSSG (Hysterocontrast-Sonography), MRI (Magnetic Resonance Imaging), TVS (Transvaginal Sonography), 2D (Two-Dimensional), 3D (Three-Dimensional), ART (Assisted Reproductive Technology).

**Table 2:** UP Patterns and adenomyosis-associated symptoms.

First Author, Year of Publication	Frequency (Contraction/min)	Direction (Study Group)	Direction (Control Group)	Adenomyosis Diagnostic	Classification	Chronic Pain/ Pelvic pain	Infertility Associated	AUB Associated	Endometriosis Associated
Kissler et al., 2006 [25]	NR	Focal: ipsi/bilateral 10/24 (42%), contralateral 8/24 (33%), failure 6/24 (25%); Diffuse: ipsi/bilateral 1/11 (9%), contralateral 2/11 (18%), failure 8/11 (73%)	ipsi/bilateral 4/6 (67%), contralateral 2/6 (33%), failure 0/6 (0%)	T2-MRI (JZ ≥ 9 mm)	NR	NR	NR	NR	35/35 (100%)
Kissler et al., 2007 [26]	NR	Focal: physiologic 13/28 (46%), pathophysiologic 15/28 (54%); Diffuse: physiologic 3/14 (21.5%), pathophysiologic 11/14 (78.5%)	Physiologic 5/8 (62.5%), pathophysiologic 3/8 (37.5%)	T2-MRI	NR	NR	NR	NR	NR
Soares et al., 2023 [27]	Adenomyosis: 0.8/2 min; Without: 3.18/2 min	1/4 (25%) antegrade; 3/5 (75%) retrograde; 11/11 (100%) control retrograde	NR	MRI	NR	NR	NR	NR	NR
Arena et al., 2024 [28]	NR	2/18 (11.1) antegrade; 5/18 (27.8) retrograde; 7/18 (38.9) opposing; 4/18 (22.2) random; 0 absent	3/18 (16.7) antegrade; 13/18 (72.2) retrograde; 1/18 (5.6) opposing; 1/18 (5.6) random; 0 absent	TVS (2D)—3D software reconstruction	MUSA	2.6 ± 3.0 vs. 0.4 ± 1.0	6 (33.3%) vs. 2 (11.1%)	9 (50%) vs. 1 (5.6%)	NR
Rees et al., 2024 [29]	Late follicular Adenomyose: 1.54 ± 0.26 Control group: 1.70 ± 0.26	Direction not explicitly reported. Velocity was measured for both F2C and C2F propagation, and differences can be interpreted, but no cut-off values were defined	Direction not explicitly reported. Velocity was measured for both F2C and C2F propagation, and differences can be interpreted, but no cut-off values were defined	TVS/MRI	MUSA	NR	NR	NR	NR
Kido et al., 2025 [30]	proliferative phase focal AM: 8.8/3 min diffuse AM: 8.06/3 min healthy: 9.52/3 min	cervix—fundus: focal AM: 11/22 diffuse AM: 19/27 fundus—cervix: focal AM: 4/22 diffuse AM: 2/27 opposing: focal AM: 5/22 diffuse AM: 3/27	cervix—fundus: 11/24 fundus—cervix: 8/24 opposing: 4/24	TVS/MRI	NR	NR	NR	NR	NR
Latif et al., 2025 [31]	Ovarian stimulation 3.03	5/26 antegrade vs. 0/40 control 7/26 opposing vs. 6/40 control 6/26 random	0 retrograde; others NR	TVS	NR	NR	NR	NR	NR

Abbreviation: NR (Not Reported), T2-MRI (T2-weighted Magnetic Resonance Imaging), JZ (Junctional Zone), MRI (Magnetic Resonance Imaging), TVS (Transvaginal Sonography), 2D (Two-Dimensional), 3D (Three-Dimensional), MUSA (Morphological Uterus Sonographic Assessment), AM (Adenomyosis) F2C (Fundus-to-Cervix), C2F (Cervix-to-Fundus), AUB (Abnormal Uterine Bleeding), UP (Uterine peristalsis).

**Table 3:** Newcastle–Ottawa quality assessment form for cohort studies.

First Author, Year	Representativeness of Exposed Cohort	Selection of Non-Exposed Cohort	Ascertainment of Exposure	Outcome Not Present at Study Start	Comparability of Cohorts (Confounders Controlled)	Assessment of Outcome	Sufficient Length of Follow-Up	Adequacy of Follow-Up of Cohorts	Total	Quality Assessment
Kissler, 2006 [25]	★	-	★	-	–	★	–	★	4	poor
Kissler, 2007 [26]	–	–	–	–	–	–	–	–	-	-
Soares, 2023 [27]	–	★	★	-	–	★	–	★	4	poor
Arena, 2024 [28]	★	★	★	-	–	★	–	★	5	fair
Rees, 2024 [29]	★	★	★	-	–	★	–	★	5	fair
Kido, 2025 [30]	★	★	★	-	–	★	–	★	5	fair
Latif, 2025 [31]	★	★	★	-	–	★	–	★	5	fair

Each star (★) in the table represents one point awarded according to the Newcastle–Ottawa Scale (NOS), which was used to assess the methodological quality of the included observational studies.

### 2.6. Data Synthesis

The primary outcome was the assessment of UP in women with and without adenomyosis. For the meta-analysis, we used the mean and SD of the frequency of the number of uterine contractions in the adenomyosis group and the control group as well as the direction of uterine contractions in women with adenomyosis compared to controls. The pooled results of standardized mean differences (SMDs) with 95% CIs in levels of uterine peristalsis were analyzed using the “metafor” and “dmetar” functions in R software (Version 4.2.3, R Core Team, Vienna, Austria, 2013). To examine heterogeneity, we used Cohen’s Q statistic and I^2^ statistic. In the presence of high heterogeneity, we employed random effects models.

### 2.7. Meta-Analysis Visualisation

Forest plots were used to visually summarize the meta-analytic results. Each horizontal line represents an individual included study with its point estimate (box) and corresponding 95% confidence interval (CI, line). The size of the box reflects the statistical weight of each study. The overall summary estimate is depicted as a diamond at the bottom of the plot, whose center and width visualize the pooled effect size and its 95% CI, respectively. The vertical line indicates the ‘line of no effect’ (OR or RR = 1; SMD or MD = 0): results to the left or right of this line indicate decreased or increased peristalsis in adenomyosis compared to controls. Estimates crossing this line are not statistically significant. Colors enhance clarity: navy for individual studies and dark green for the pooled estimate. Studies with statistically significant results (*p* < 0.05) are colored in red.

## 3. Results

### 3.1. Results of the Systematic Review

After screening the abstracts and the full text of the study topic, 611 studies remained. However, we excluded 605 of these studies as they did not fit our predetermined inclusion criteria. Therefore, 7 articles were included in the final systematic review (Figure 1).

### 3.2. Study Characteristics

The characteristics of the study populations are summarized in Table 1. The 7 studies were prospective. Patients included in the 7 studies [25,26,27,28,29,30,31] were predominantly European (5 studies), with one study conducted in Japan and one in Brazil. A total of 442 women were included in the review. Between 109 and 219 women (32%) were eligible for subgroup analysis, which was the basis for the meta-analysis. Although Kissler et al. (2007) [26] was a review, it was included as it reported additional patients beyond the 2006 dataset, thereby enabling analysis of a larger cohort. Study sample sizes varied from 36 to 145 patients. The methodological quality of the studies was rated mainly fair (n = 4) and poor (n = 2), primarily due to the absence of a comparison group (Table 3).

### 3.3. Results of Individual Studies

Interpretation of the findings required analysis of uterine peristalsis, taking into account different parameters such as frequency, amplitude, and direction (Table 2). Moreover, the measurements comprised different phases of the menstrual cycle.

#### 3.3.1. Frequency of Uterine Contractions

Concerning the frequency of uterine contractions, studies demonstrate contradictory patterns between adenomyosis, resulting in divergent conclusions regarding the impact of these conditions on patients’ reproductive function [28,29,30]. Specifically, in adenomyosis, the UP frequency pattern along the menstrual cycle is often diminished and disorganised, particularly during the follicular phase and in women experiencing severe dysmenorrhea. Furthermore, this decrease in frequency is accompanied by a lack of coordination of peristaltic movements, suggesting an overall alteration in uterine dynamics [28,30].

#### 3.3.2. Amplitude of Uterine Contractions

The amplitude of uterine contractions is a key consideration in the diagnosis of adenomyosis. A progressive increase in this parameter is observed in cases of severe dysmenorrhea and during the late follicular and luteal phases, accompanied by a decrease in frequency and velocity [29]. This finding suggests the presence of a pattern characterized by more intense contractions, albeit less coordinated.

#### 3.3.3. Direction of Uterine Contractions

An altered direction of uterine contractions is indicative of adenomyosis, which exhibits a random and disorganized pattern with a higher proportion of retrograde movements. Retrograde movements have been associated with pelvic pain and menstrual disorders. The abnormal direction is primarily characterized by an increase in cervico-fundal contractions during the follicular phase and a high rate of retrograde flow, which is considerably higher than that observed in women without the disease.

#### 3.3.4. Association with Symptoms of Adenomyosis

Associated clinical symptoms include hyperfrequency and hyperamplitude of contractions, as well as intensity of pelvic pain and dysmenorrhea [11,18]. Furthermore, hyperperistalsis, dyspermia, and abnormal uterine bleeding have been identified as pathophysiological mechanisms that impede fertility by interfering with sperm transport and embryo implantation. Accordingly, the existent evidence corroborates the hypothesis that alterations in uterine contractile dynamics represent a pivotal component of the pathophysiology and clinical expression of these pathologies.

### 3.4. Results of the Meta-Analysis

#### Uterine Contraction Frequency

**Figure 2:**
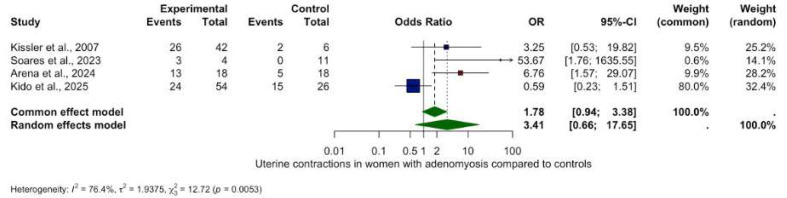
Uterine contractions in women with and without adenomyosis [26,27,28,30]. Forest plot of odds ratios (OR) and 95% confidence intervals (CI) for studies evaluating UP in women with and without adenomyosis. The blue squares in each study indicate the OR, the size of the squares indicates the study weight, and the horizontal lines indicate the 95% CI. Overall estimates are presented in the fixed- and random-effects models. The prediction interval is defined as the interval within which the effect size of a new study would fall if this study were selected at random from the same population of studies already included in the meta-analysis.

Four studies (219 women) provided data for frequency analysis. Women with adenomyosis suggest significantly reduced uterine contraction frequency compared to controls (SMD −1.81, 95% CI: −3.04 to −0.58, *p* = 0.0039) (Figure 2). However, substantial heterogeneity was observed (I^2^ = 82.0%, *p* = 0.0008), indicating considerable variation between studies.

### 3.5. Directional Patterns of Uterine Contractility

#### 3.5.1. Antegrade Uterine Contractions

The only statistically significant directional finding was a reduction in antegrade contractions in women with adenomyosis (OR 0.35, 95% CI: 0.13–0.96, *p* = 0.0423) with low heterogeneity (I^2^ = 0.0%).

#### 3.5.2. Other Contractility Patterns

**Figure 3:**
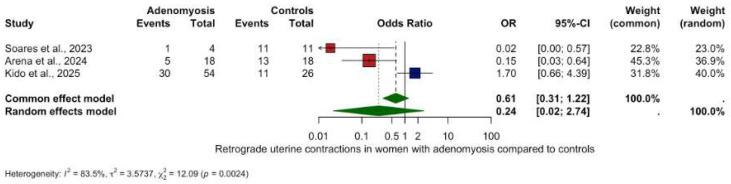
Retrograde uterine contractions in women with and without adenomyosis [27,28,30]. For details see legend of Figure 2.

**Figure 4:**
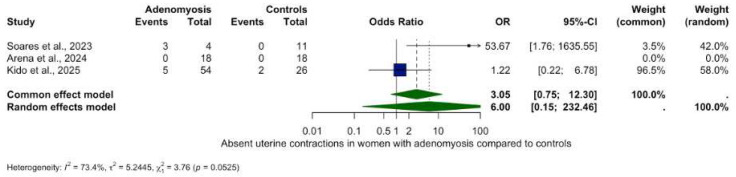
Absent uterine contractions in women with and without adenomyosis [27,28,30]. For details see legend of Figure 2.

**Figure 5:**
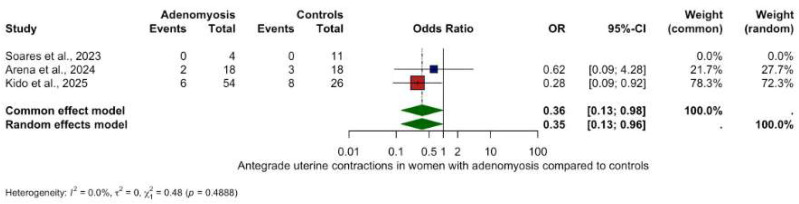
Antegrade uterine contractions in women with and without adenomyosis [27,28,30]. For details see legend of Figure 2.

**Figure 6:**
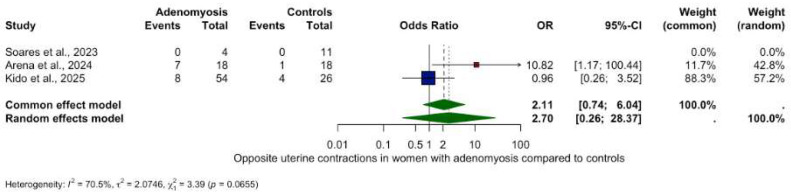
Opposite uterine contractions in women with and without adenomyosis [27,28,30]. For details see legend of Figure 2.

Analysis of other contractility patterns revealed non-significant trends only:

1. Retrograde contractions: OR 0.24 (95% CI: 0.02–2.74, *p* = 0.2534), (Figure 3).
2. Absent contractions: OR 6.00 (95% CI: 0.15–232.46, *p* = 0.3370), (Figure 4).
3. Antegrade contractions: OR 3.50 (95% CI: 0.15–0.96, *p* = 0.048), (Figure 5).
4. Opposite contractions: OR 2.70 (95% CI: 0.26–28.37, *p* = 0.4079), (Figure 6).
5. Random contractions: OR 4.32 (95% CI: 0.73–25.46, *p* = 0.1063).

All these analyses showed substantial heterogeneity (I^2^ > 70%), limiting the reliability of pooled estimates. The small number of available studies also prevents a reliable assessment of publication bias, which therefore cannot be excluded.

## 4. Discussion

The aim of the present systematic review was to evaluate the association of UP disruption in patients with uterine adenomyosis compared to controls. Our findings provide limited but significant evidence of altered uterine contractility in adenomyosis. Our findings demonstrate two key significant alterations: reduced overall contraction frequency and decreased antegrade contractions in women with adenomyosis compared to controls. Importantly, many previously suggested patterns (increased retrograde contractions, absent contractions) were not statistically significant in our pooled analysis, highlighting the need for caution in clinical interpretation.

Adenomyosis is defined by the ectopic localization of endometrial glands and stroma within the myometrium, resulting in thickening of the junctional zone. Although the eutopic endometrium may appear morphologically unremarkable, evidence indicates that its receptivity is functionally compromised in adenomyosis [32,33]. The prevalence of adenomyosis, a condition that affects women of reproductive age, has been observed to range between 20% and 25% [6,34]. Mechanisms linking infertility to adenomyosis have been identified. Several hypotheses have been proposed to explain this association. The potential etiologies encompass a range of factors, including impaired implantation due to altered endometrial receptivity, involvement of the junctional zone, estrogen dominance, progesterone resistance, immunological alterations, and uterine hypercontractility [15]. This distortion of contractility exerts a detrimental effect on reproductive outcomes, manifesting not only in the follicular phases, but also in the luteal phase, which encompasses embryo implantation and pregnancy maintenance [10,11,25].

**Figure 7:**
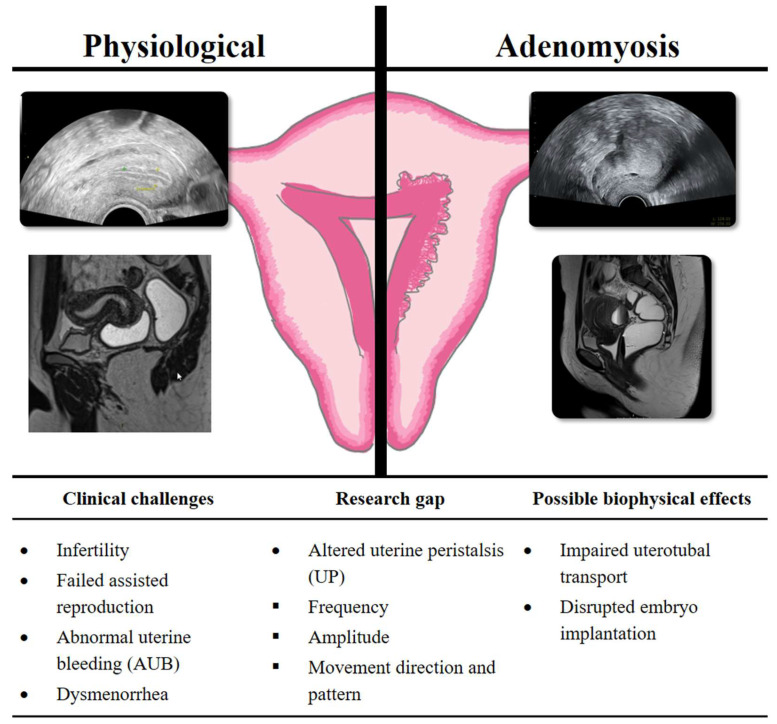
Impact of adenomyosis on uterine physiology, clinical challenges, and biophysical effects.

However, uterine peristalsis is a crucial biomechanical activity for fertilization and implantation [11,12,13]. The rhythmic contractions of the uterus during the menstrual cycle exhibit significant physiological variations in terms of frequency, amplitude, and direction described from van Gestel et al. (2003) [35]. Prior to the occurrence of ovulation, an increase in the frequency of uterine contractions directed caudally has been observed, thereby facilitating the transport of sperm to the fallopian tubes [11]. Such changes in the direction of the undulating movements are caused by the production of local hormones. Within the luteal phase, the uterus undergoes hypokinetic changes that create a favorable environment for embryo implantation [36,37]. Therefore, exploring this contractile activity in patients with gynecological diseases is important to understand the effect on the different parameters of uterine peristalsis in the different phases of the cycle, as well as to establish the significance of this contractile activity in such contexts (Figure 7) [21,38].

Another significant aspect to consider is the correlation between endometriosis and adenomyosis [4]. A high prevalence of association has been observed between adenomyosis and endometriosis, suggesting that they may manifest as a closely related disorder with remarkably similar pathophysiology. Uterine contractility is characterized by irregularity in patients with endometriosis, which may promote its progression through retrograde menstruation [10,39] (Supplementary File S2, Uterine Contractility Video). Furthermore, abnormal uterine contractility is a mechanical factor contributing to infertility [38]. The results of the meta-analysis conducted highlight the relevance of abnormal uterine contractility activity and its correlation with endometriosis.

To facilitate a more profound comprehension of the subject, it is imperative to elucidate the concepts of uterine peristalsis and calcium (Ca^2+^) oscillations [40]. Uterine peristalsis is a process that is closely linked to Ca^2+^ oscillations in circular smooth muscle [15]. Under physiological conditions, uterine contractions manifest as coordinated patterns associated with Ca^2+^ oscillations [41]. However, in the case of adenomyosis, this pattern is altered, manifesting as isolated peaks of activity, of lower intensity and with more attenuated Ca^2+^ signals, although without a significant variation in the total frequency [42]. Such a change in the mode of contraction suggests that the determining factor is not the number of contractions, but rather the method in which they occur [41,42]. This hypothesis was confirmed by the results of the present study, which demonstrated that adverse UP plays a crucial role in the pathophysiology of clinical manifestations in adenomyosis and helps explain resulting problems such as infertility, dysmenorrhea and chronic pelvic pain.

The immunological standpoint suggests that adenomyosis is associated with the presence of CE (chronic endometritis), indicating chronic inflammation resulting in hyperperistalsis [43,44,45,46]. The two conditions under discussion have been demonstrated to exhibit characteristics of chronic inflammation, including elevated local concentrations of proinflammatory cytokines, such as TNF-α and IL-6 [44,47]. These have been demonstrated to disrupt the coordinated peristaltic activity of the uterus. Alterations to the junctional zone, a hallmark of adenomyosis, have the capacity to modify the interface between the endometrium and myometrium [48]. Consequently, this disruption may contribute to the persistence of microbial pathogens and the progression of CE [49]. Future studies should further investigate this possible association, ideally through well-designed prospective studies using uniform diagnostic criteria and controlling for confounding factors such as coexisting endometriosis.

Our findings are consistent with those of recent studies on UP, which also reported decreased contraction coordination in women with adenomyosis compared to controls. Consequently, MUSA criteria must be considered when evaluating the impact of varying degrees of adenomyosis [50,51]. In our review, only two studies used MUSA criteria to define adenomyosis [28,29]. A better understanding of the pathophysiology of infertility with associated adenomyosis, can be achieved through accurate diagnosis and classification of adenomyosis.

Despite the study’s meticulous adherence to the guidelines for the preparation of concise, high-quality reports on the evidence, it is important to acknowledge the limitations of the study and consider these in future research. Firstly, it should be noted that the number of studies and patients included in the analysis was limited, as only 7 studies met the inclusion criteria. Secondly, the methodology employed in the reviewed studies exhibits a paucity of scientific rigor, consequently yielding an absence of pertinent information concerning the classification of adenomyosis. Thirdly, it is crucial to examine the lack of information on UP at various stages of the cycle in order to optimize its interpretation. The substantial heterogeneity observed across most analyses (I^2^ > 70%) limits the robustness of our conclusions. This variability likely reflects differences in imaging modalities, menstrual phases at the time of assessment, and diagnostic criteria applied for adenomyosis, as well as variations in measurement techniques and patient populations. Exploring these factors may help to explain the observed inconsistencies and should be a focus of future research. Notably, only the analysis of antegrade contractions demonstrated acceptable heterogeneity (I^2^ = 0%), supporting this as the most consistent finding in our study.

Future studies should concentrate on formulating precise diagnostic criteria for the identification of adenomyosis subtypes. The standardization of uterine contractility measurement is a critical aspect of research on its impact on embryo implantation and pregnancy rates in assisted reproductive treatments (Figure 7). The implementation of advanced techniques, such as 3D and 4D transvaginal ultrasound, along with emerging tools like electrohysterography and AI (artificial intelligence) would allow for more reliable and reproducible data, facilitating the integration of uterine contractility as a key factor in clinical practice. Additionally, one prospective trial registered on BASEC (“EndoAdenoUpistalsis”, Nr: 2024-01617) is currently investigating the impact of endometriosis and adenomyosis on uterine peristalsis in stimulated early cycle and luteal phase and might be able to demonstrate that electrohysterography (EHG) as well as 4D ultrasound could serve as reference tools for measuring uterine activity in the field of reproductive medicine.

In conclusion, this systematic review and meta-analysis provides significant evidence for two specific alterations in uterine contractility associated with adenomyosis: reduced contraction frequency and decreased antegrade contractions. However, the substantial heterogeneity in most analyses and small number of included studies limit the clinical applicability of these findings. Many previously hypothesized contractility patterns were not statistically confirmed. A more profound comprehension of this phenomenon in healthy conditions and adenomyosis is needed for advancements to be made in our understanding of reproductive physiology Future research requires standardized diagnostic criteria, larger sample sizes, and consistent measurement protocols to provide definitive evidence for clinical decision-making.

## Data Availability

The original contributions presented in this study are included in the article and Supplementary Materials. Further inquiries can be directed to the corresponding author.

## Supplementary Materials

The following supporting information can be downloaded at: https://www.mdpi.com/article/10.3390/biomedicines13112728/s1, Supplementary File S1: Database Searching Strategies. Systematic literature search in Medline, Embase, Cochrane, CENTRAL and Google Scholar. Supplementary File S2: Video showing the uterine contractility in the presence of adenomyosis.

## Abbreviations

AI	Artificial Intelligence
ART	Assisted Reproductive Technology
Ca^2+^	Calcium ion
CE	Chronic Endometritis
CENTRAL	Cochrane Central Register of Controlled Trials
CI	Confidence Interval
CPR	Clinical Pregnancy Rate
EHG	Electrohysterography
IVF	In Vitro Fertilization
JZ	Junctional Zone
MD	Mean Difference
MRI	Magnetic Resonance Imaging
MUSA	Morphological Uterus Sonographic Assessment
NOS	Newcastle–Ottawa Scale
OR	Odds Ratio
PRISMA	Preferred Reporting Items for Systematic Reviews and Meta-Analyses
PROSPERO	International Prospective Register of Systematic Reviews
SD	Standard Deviation
TVS	Transvaginal Sonography
UP	Uterine Peristalsis
2D/3D/4D	Two-/Three-/Four-Dimensional (Ultrasound)
