# Supplementary material for: Uterine Contractility Changes in Adenomyosis: Evidence from a Systematic Review and Meta-Analysis
Source: Biomedicines. 2025 Nov 6;13(11):2728. doi: 10.3390/biomedicines13112728 (PMC12650493; doi:10.3390/biomedicines13112728)
Supplement: Supplementary file 1 [file biomedicines-13-02728-s001.zip › File S1_uterineperistalsis_adenomyosis_searchdocu.pdf]

Uterine peristalsis in adenomyosis

- METHODS -

We designed a systematic literature search strategy focusing on the following concepts: (1) uterine peristalsis, and (2) adenomyosis.

The searches were conducted in MEDLINE, Embase, Scopus, the Cochrane Central Register of Controlled Trials (CENTRAL), and the Google Scholar platform.

An initial search strategy was developed by an information specialist in Embase and validated against a set of key references to ensure the inclusion of relevant core publications. Following iterative refinement, tailored search strategies were constructed for each database, incorporating both controlled vocabulary (e.g., MeSH, Emtree) and free-text terms. The free-text component included synonyms, acronyms, and related terminology.

In MEDLINE, Embase, and CENTRAL, animal-only studies were excluded using a double-negative filter based on MeSH and Emtree terms (Ovid 'humans only' filter[1]). In Scopus, studies were limited to human research using the built-in filters based on keywords for animals, which were excluded.

Systematic reviews and meta-analyses were excluded by applying the relevant publication type data field in MEDLINE, and by excluding such studies through the title field in Embase and Scopus.

In Google Scholar, results were limited to the 200 most relevant records, including citations, and were exported using Harzing's Publish or Perish software[2].

The search was completed on June 17, 2025.

Duplicate records were primarily removed using the deduplication tool Deduklick[3,4], followed by manual cleaning in EndNote[5]. Screening took place in Covidence[6].

---

[1] Ovid MEDLINE and Embase Database Guides 2025:
https://ospguides.ovid.com/OSPguides/medline.htm and
https://ospguides.ovid.com/OSPguides/embase.htm
[2] Harzing, A.W. (2007) Publish or Perish, available from https://harzing.com/resources/publish-or-perish
[3] https://risklick.ch/panel/help/systematic-review/deduklick
[4] Borissov, Nikolay et al. "Reducing systematic review burden using Deduklick: a novel, automated, reliable, and explainable deduplication algorithm to foster medical research." Systematic reviews vol. 11,1 172. 17 Aug. 2022, doi:10.1186/s13643-022-02045-9
[5] https://endnote.com/
[6] https://www.covidence.org/

- OVERVIEW -

Bibliographic Databases					
**Search date**	**Database searched**	**Platform / Provider**	**Dates of database coverage**	**Records**	**Records after deduplication**
17 June 2025	MEDLINE ALL	Ovid (Wolters Kluwer)	1946 – June 16, 2025	159	158
17 June 2025	Embase	Ovid (Wolters Kluwer)	1946 – June 16, 2025	299	149
17 June 2025	Scopus	Elsevier	1788 - present	308	112
17 June 2025	Cochrane CENTRAL Database of Clinical Trials	Wiley	1908 - present	15	9
17 June 2025	Google Scholar	Google via Harzing's Publish or Perish	To present	200	184
**Sum of references**				981	**612**

- SEARCH STRATEGIES -

MEDLINE/Ovid

Search date: 17 June 2025

1 exp Uterine Contraction/ 7966

2 exp Uterus/ 129584

3 exp Peristalsis/ 4259

4 2 and 3 48

5 (((uter* or junctional zone* or myometri* or endometri* or intrauter* or intra uter* or wall) adj4 (contract* or peristal* or dynamic* or activit* or relax* or wave* or motil* or hypercontract* or hyper contract*)) or contract* frequen* or hyperperistals* or dysperistals*).ti,ab,kf. 26299

6 1 or 4 or 5 29331

7 exp Adenomyosis/ 1580

8 (adenomyo* or endometrial invasion* or endometrial infiltrat* or (ectopic endometrium adj2 myometrium) or internal endometrios* or (hypertroph* adj3

myometri*) or endometrial adenoma* or (endometriosis adj3 uterin*) or stromal endometrios*).ti,ab,kf. 6824

9 7 or 8 6866

10 6 and 9 172

11 (exp animals/ or exp animal experimentation/ or exp models, animal/ or exp plants/ or exp fungi/) not humans/ 5809822

12 10 not 11 162

13 (meta analysis or "systematic review").pt. 382912

14 12 not 13 159

Link to search:
https://ovidsp.ovid.com/ovidweb.cgi?T=JS&NEWS=N&PAGE=main&SHAREDSEARCHID=61wIlSVVVyXpCC2ifughjWWj4TTvslkcEYHFLIAKScoHysVIg7YPSuHYjmnXsl1HH

****

Embase/Ovid
Search date: 17 June 2025

1 exp uterus contractility/ 3726

2 (((uter* or junctional zone* or myometri* or endometri* or intrauter* or intra uter* or wall) adj4 (contract* or peristal* or dynamic* or activit* or relax* or wave* or motil* or hypercontract* or hyper contract*)) or contract* frequen* or hyperperistals* or dysperistals*).ti,ab,kf. 32441

3 1 or 2 33481

4 exp adenomyosis/ 8360

5 (adenomyo* or endometrial invasion* or endometrial infiltrat* or (ectopic endometrium adj2 myometrium) or internal endometrios* or (hypertroph* adj3 myometri*) or endometrial adenoma* or (endometriosis adj3 uterin*) or stromal endometrios*).ti,ab,kf. 10225

6 4 or 5 12685

7 3 and 6 328

8	(exp animal/ or exp invertebrate/ or nonhuman/ or animal experiment/ or animal tissue/ or animal model/ or exp plant/ or exp fungus/) not (exp human/ or human tissue/)	8314850

9	7 not 8	308

10	(systematic review* or meta analys* or metaanalys*).ti.	465428

11	9 not 10	299

Link to search:
https://ovidsp.ovid.com/ovidweb.cgi?T=JS&NEWS=N&PAGE=main&SHAREDSEARCHID=5q4o07kH2KUbylynI6KbnmEO8Su9P2ie0STtwL8CqOyNdIVkDq8ddECxHkwgTr5c0

****

Scopus
Search date: 17 June 2025

((TITLE-ABS-KEY (((uter* OR "junctional zone*" OR myometri* OR endometri* OR intrauter* OR "intra uter*" OR wall) W/4 (contract* OR peristal* OR dynamic* OR activit* OR relax* OR wave* OR motil* OR hypercontract* OR "hyper contract*")) OR "contract* frequen*" OR hyperperistals* OR dysperistals*)) AND (TITLE-ABS-KEY (adenomyo* OR "endometrial invasion*" OR "endometrial infiltrat*" OR ("ectopic endometrium" W/2 myometrium) OR "internal endometrios*" OR (hypertroph* W/3 myometri*) OR "endometrial adenoma*" OR (endometriosis W/3 uterin*) OR "stromal endometrios*"))) AND NOT (TITLE ("systematic review*" OR "meta analys*" OR metaanalys*)) AND (EXCLUDE (EXACTKEYWORD , "Animals") OR EXCLUDE (EXACTKEYWORD , "Animal") OR EXCLUDE (EXACTKEYWORD , "Mouse") OR EXCLUDE (EXACTKEYWORD , "Animal Model") OR EXCLUDE (EXACTKEYWORD , "Rat"))

Results: 308

****

CENTRAL
Search date: 17 June 2025

#1	[mh "Uterine Contraction"] OR ([mh Uterus] AND [mh Peristalsis])	459

#2	(((uter* OR ("junctional" NEXT zone*) OR myometri* OR endometri* OR intrauter* OR ("intra" NEXT uter*) OR wall:ti,ab,kw) NEAR/4 (contract* OR peristal* OR dynamic*

OR activit* OR relax* OR wave* OR motil* OR hypercontract* OR ("hyper" NEXT contract*):ti,ab,kw)) OR (contract* NEXT frequen*) OR hyperperistals* OR dysperistals*):ti,ab,kw 3408

#3 #1 OR #2 3408

#4 [mh Adenomyosis] 77

#5 (adenomyo* OR ("endometrial" NEXT invasion*) OR ("endometrial" NEXT infiltrat*) OR ("ectopic endometrium" NEAR/2 myometrium) OR ("internal" NEXT endometrios*) OR (hypertroph* NEAR/3 myometri*:ti,ab,kw) OR ("endometrial" NEXT adenoma*) OR (endometriosis NEAR/3 uterin*) OR ("stromal" NEXT endometrios*)):ti,ab,kw 611

#6 #4 OR #5 611

#7 #3 AND #6 15

#8 ([mh animals] OR [mh "animal experimentation"] OR [mh "models, animal"] OR [mh plants] OR [mh fungi]) NOT [mh ^humans] 3510

#9 #7 NOT #8 in Trials 15

Link to search:
https://www.cochranelibrary.com/advanced-search/search-manager;jsessionid=439BDFCD7A068EA731F3F31780981C73?search=7746271

****

Google Scholar
Search date: 17 June 2025

(uterus OR uterine OR "junctional zone" OR myometrium OR endometrium OR intrauterine OR intra uterine OR "uterine wall") AND (contraction OR peristalsis OR activity OR "relaxation" OR wave OR motility OR hypercontraction" OR contractility OR hyperperistalsis OR dysperistalsis)

AND

(adenomyosis OR adenomiotic OR "endometrial invasion" OR "endometrial infiltration" OR "ectopic endometrium" OR myometrium OR "internal endometriosis" OR (hypertrophy AND myometrium) OR "endometrial adenoma" OR (endometriosis AND uterine) OR "stromal endometriosis")

Maximum results: 200, Include Citations
